# Multi-scale asynchronous correlation and 2D convolutional autoencoder for adolescent health risk prediction with limited fMRI data

**DOI:** 10.3389/fncom.2024.1478193

**Published:** 2024-10-15

**Authors:** Di Gao, Guanghao Yang, Jiarun Shen, Fang Wu, Chao Ji

**Affiliations:** ^1^School of Physical Education, China University of Mining & Technology (Beijing), Beijing, China; ^2^Physical Education Teaching and Research Section, Beijing City University, Beijing, China

**Keywords:** adolescence, health risk assessment, functional magnetic resonance imaging, deep learning, convolutional autoencoder, behavioral health risks

## Abstract

**Introduction:**

Adolescence is a fundamental period of transformation, encompassing extensive physical, psychological, and behavioral changes. Effective health risk assessment during this stage is crucial for timely intervention, yet traditional methodologies often fail to accurately predict mental and behavioral health risks due to the intricacy of neural dynamics and the scarcity of quality-annotated fMRI datasets.

**Methods:**

This study introduces an innovative deep learning-based framework for health risk assessment in adolescents by employing a combination of a two-dimensional convolutional autoencoder (2DCNN-AE) with multi-sequence learning and multi-scale asynchronous correlation information extraction techniques. This approach facilitates the intricate analysis of spatial and temporal features within fMRI data, aiming to enhance the accuracy of the risk assessment process.

**Results:**

Upon examination using the Adolescent Risk Behavior (AHRB) dataset, which includes fMRI scans from 174 individuals aged 17–22, the proposed methodology exhibited a significant improvement over conventional models. It attained a precision of 83.116%, a recall of 84.784%, and an F1-score of 83.942%, surpassing standard benchmarks in most pertinent evaluative measures.

**Discussion:**

The results underscore the superior performance of the deep learning-based approach in understanding and predicting health-related risks in adolescents. It underscores the value of this methodology in advancing the precision of health risk assessments, offering an enhanced tool for early detection and potential intervention strategies in this sensitive developmental stage.

## 1 Introduction

Adolescence is a critical period of individual development, during which significant physiological, psychological, and social changes occur, profoundly impacting long-term health outcomes (Su et al., 2020; Tate et al., 2020). However, adolescents face increasing health risks, including mental health issues such as depression and anxiety, behavioral problems like substance abuse and violent tendencies, as well as other health-related behaviors such as eating disorders and lack of physical activity (Bozzini et al., 2020; Zink et al., 2020; Scardera et al., 2020). Early identification and intervention of these risks are crucial for safeguarding the future health of adolescents. In recent years, the

development of functional magnetic resonance imaging (fMRI) technology has provided powerful tools for studying the neural changes in adolescents and their relationship with health risks (Baranger et al., 2021; Lee et al., 2023; Sripada et al., 2020). However, acquiring and annotating high-quality fMRI data is often challenging due to the high costs and technical expertise required, limiting its use in large-scale population studies. Moreover, the rise of deep learning and machine learning technologies has further advanced health risk assessment research based on fMRI data. By combining these technologies, researchers can more accurately analyze brain function data in adolescents, predict their health risks, and provide valuable support for clinical decision-making. To address the challenges of limited fMRI data, autoencoders have been introduced to reconstruct and reduce the dimensionality of fMRI data, thereby reducing the cost and improving the efficiency of model building.

Functional magnetic resonance imaging (fMRI) is widely used in neuroscience research as a non-invasive imaging technique that captures brain activity by detecting blood oxygen level-dependent (BOLD) signals (Lauharatanahirun et al., 2023; Agarwal et al., 2023). This allows researchers to study the activity patterns of different brain regions under specific tasks or psychological states, revealing the neural mechanisms associated with health risks. However, the application of fMRI technology faces several challenges. First, the high cost of acquiring and processing fMRI data limits its use in large-scale population studies. Second, fMRI is sensitive to noise and individual differences, requiring careful interpretation of the data, often necessitating additional data sources and expert judgment (Viessmann and Polimeni, 2021; Bollmann and Barth, 2021). Additionally, the scanning process may cause discomfort for some participants, particularly adolescents, potentially impacting data reliability.

To address these issues, researchers have begun applying deep learning and machine learning models to fMRI data analysis. CNNs can automatically extract complex spatial features from fMRI images, improving the efficiency and accuracy of feature extraction without relying on traditional manual feature selection methods (Yin et al., 2022; Lin et al., 2022). Autoencoders, on the other hand, achieve dimensionality reduction and denoising through unsupervised learning, reducing redundant information, and enhancing model robustness (Kim et al., 2021; Qiang et al., 2021). Despite the significant advantages these models offer in fMRI data analysis, they still face certain limitations. Firstly, deep learning models typically require large amounts of labeled data for training, and the high cost of acquiring and labeling fMRI data limits the size of the training datasets. Secondly, the “black box” nature of deep learning models makes them difficult to interpret, which is particularly important in medical applications (Sheu, 2020). Additionally, issues such as overfitting and high computational complexity may limit the performance of these models in practical applications.

Despite the potential demonstrated by the integration of fMRI technology with deep learning models in health risk assessment, there are still several challenges in practical application. Firstly, developing efficient and accurate models with limited data remains a pressing issue (Allen et al., 2022). Secondly, improving the interpretability of the models is crucial for enabling clinicians to understand and trust the predictions made by these models. Moreover, the lack of standardized assessment methods and criteria makes it difficult to generalize these models across different populations and settings. Therefore, the motivation of this paper is to develop an adolescent health risk assessment method that combines fMRI and deep learning. This method aims to efficiently extract and analyze brain function data while improving the accuracy and interpretability of predictions, thereby better serving the health management of adolescent populations.

This paper proposes a method for adolescent health risk prediction that integrates multi-sequence two-dimensional convolutional autoencoders (2DCNN-AE) with multi-scale asynchronous correlation information extraction. Initially, raw fMRI data is preprocessed using the PyReliMRI toolkit, including head motion correction, slice timing correction, and spatial normalization. The 2DCNN-AE model is then employed to extract spatial and temporal features from the preprocessed fMRI data. This model consists of an encoder and a decoder, where the input data is feature-encoded and reconstructed through convolutional layers and upsampling layers. Additionally, a multi-sequence and multi-scale asynchronous correlation information extraction method is introduced, mapping brain partition maps under three-dimensional spatial coordinates to specific brain functional areas and extracting the probability distribution of synchronous expression between different time series. Finally, the extracted multi-scale asynchronous correlation information is used as feature inputs to train and construct the adolescent health risk prediction model.

The contributions of this paper are as follows:

- The proposed multi-sequence two-dimensional convolutional autoencoder (2DCNN-AE) method efficiently extracts spatial and temporal features from fMRI data, significantly improving the efficiency and accuracy of feature extraction.
- By introducing a multi-scale asynchronous correlation information extraction technique, the proposed method captures complex temporal relationships between different brain regions, thereby enhancing the robustness and predictive capability of the health risk assessment model.
- The use of autoencoders allows for the reconstruction of fMRI data samples, reducing the cost and challenges associated with acquiring large-scale annotated datasets, and thereby making the model more feasible for practical applications.
- The proposed method not only enhances the accuracy of model predictions but also improves model interpretability, making the predictions easier to understand and apply in clinical practice.

## 2 Related work

### 2.1 Functional magnetic resonance imaging techniques

The application of functional magnetic resonance imaging (fMRI) in adolescent health risk assessment represents a cutting-edge advancement in this field. By detecting blood oxygen level-dependent (BOLD) signals, fMRI indirectly reflects brain activity, offering rich spatial and temporal information (Stiernman et al., 2021). This technology is extensively used to study neurodevelopmental processes in adolescents and to identify brain function characteristics related to mental health issues, such as depression, anxiety, and attention deficit hyperactivity disorder (ADHD) (Wang et al., 2023; McNorgan et al., 2020). For example, fMRI can help pinpoint brain activity patterns associated with these common adolescent mental health challenges.

However, despite the non-invasive nature and high spatial resolution of fMRI, there are certain limitations in its practical application. First, the high cost of fMRI data acquisition and analysis restricts its use in large-scale studies. Second, fMRI is sensitive to noise and individual differences, which necessitates careful interpretation of the results (Uyulan et al., 2023). Additionally, the fMRI scanning process may cause discomfort in some participants, especially adolescents, potentially affecting the accuracy of the data. Therefore, improving data quality while reducing participant discomfort, as well as simplifying the complexity of fMRI data processing, remain significant challenges in this area.

### 2.2 Deep learning in network magnetic resonance imaging techniques

With the advancement of deep learning, researchers have increasingly integrated it with fMRI data to enhance the precision and efficiency of health risk assessment (Liu et al., 2022). Deep learning models, particularly convolutional neural networks (CNNs) and Long Short-Term Memory network (LSTM) (Saurabh and Gupta, 2024), have shown immense potential in processing and analyzing fMRI data. CNNs, with their hierarchical structure, can effectively extract complex spatial features from fMRI images (Chen et al., 2020), while AEs use unsupervised learning to achieve data dimensionality reduction and reconstruction, thereby alleviating the computational burden associated with high-dimensional data.

The application of deep learning in fMRI data analysis offers several significant advantages. For instance, CNNs can automatically extract brain activity features related to adolescent mental health risks without relying on traditional manual feature selection methods. This not only improves the efficiency of feature extraction but also captures a wider range of potential brain function patterns. Additionally, AEs excel in denoising and feature selection, making the models more robust and stable when handling fMRI data.

However, the application of deep learning in network magnetic resonance imaging also faces challenges. First, deep learning models typically require large amounts of labeled data for training, and the high cost of acquiring and labeling fMRI data limits the scale of training datasets. Second, the “black box” nature of deep learning models makes them difficult to interpret, which is particularly important in medical applications (Iravani et al., 2021). Moreover, issues such as overfitting and high model complexity may lead to suboptimal performance in practical applications. Therefore, balancing model complexity and interpretability, and training efficient models on small sample datasets, remain key areas of focus in this field.

### 2.3 Adolescent health risk assessment criteria

Adolescent health risk assessment criteria are a critical application area for combining fMRI techniques with deep learning (Ernst et al., 2015). Existing health risk assessment standards are typically based on a variety of factors, including biomarkers, behavioral assessments, and psychological questionnaires, providing essential tools for identifying at-risk adolescents (Bjork et al., 2010). However, traditional assessment methods often rely on expert judgment, which can introduce bias and inconsistency.

In recent years, researchers have sought to develop health risk assessment criteria based on fMRI data and deep learning models. For example, some studies have utilized deep learning models to automatically analyze fMRI data, extracting features related to health risks and predicting individual mental health risks based on these features (Mueller et al., 2010). This approach not only improves the objectivity and accuracy of assessments but also enables early detection of potential health issues, providing critical information for intervention and treatment.

The integration of fMRI and deep learning technologies provides powerful tools for health risk prediction. Despite the significant potential demonstrated by current research and applications, challenges remain in terms of data acquisition, model interpretability, and the standardization of assessment criteria. Future research should focus on developing more efficient, accurate, and scalable health risk assessment models to better serve the health management needs of adolescent populations.

## 3 Method

**Figure 1:**
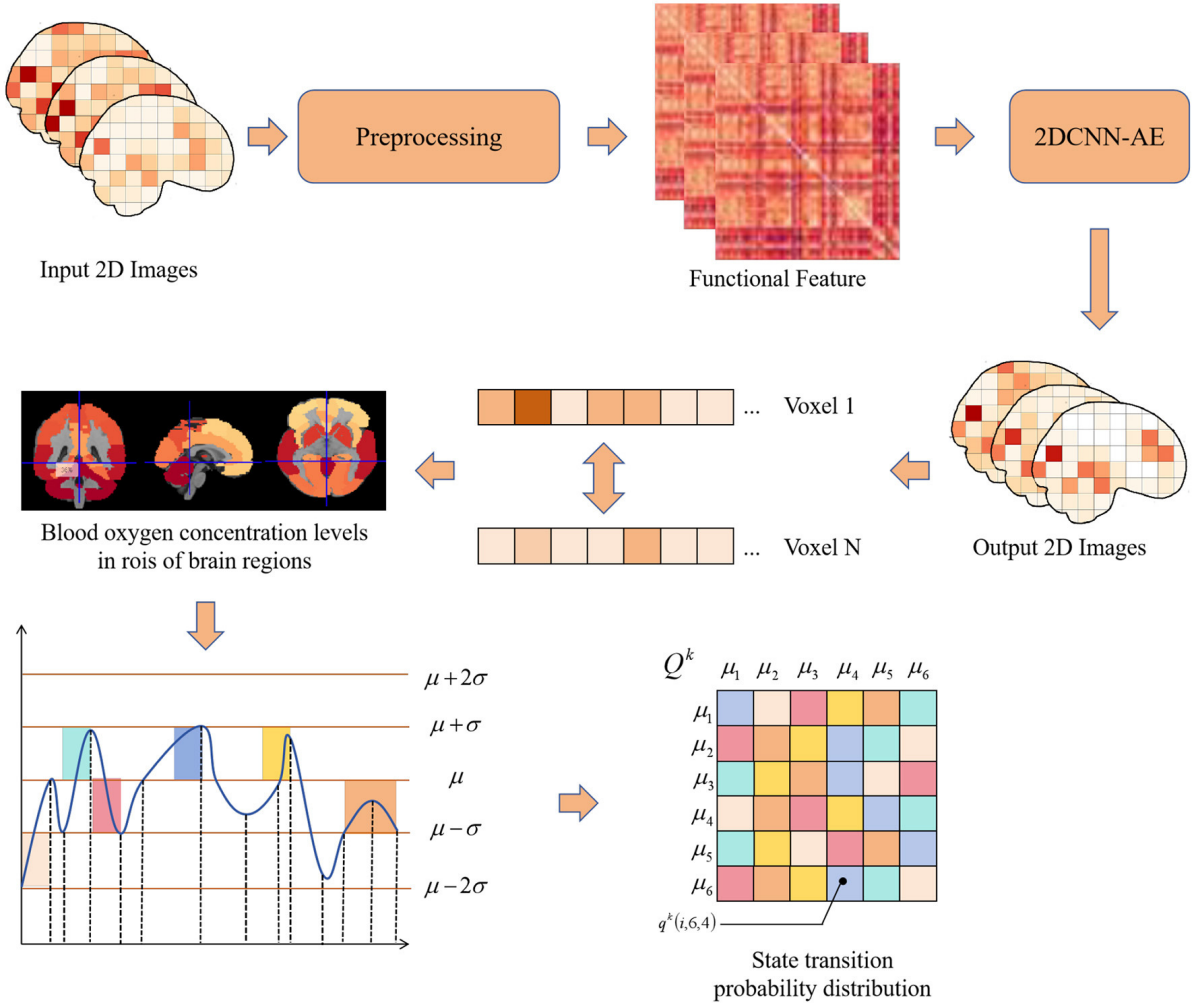
Flow chart of multiple sequence asynchronous correlation algorithm for 2D convolutional auto-encoding.

This paper proposes a method for predicting adolescent health risks by combining multi-sequence, two-dimensional convolutional autoencoder (2DCNN-AE) and multi-scale asynchronous correlation information extraction. The algorithm flow of two-dimensional convolutional autoencoder and multi-sequence asynchronous correlation is shown in Figure 1. First, the original fMRI data was preprocessed using the PyReliMRI toolkit, including head motion correction, slice timing correction and spatial normalization. Next, the 2DCNN-AE model was used to extract spatial and temporal features from the preprocessed fMRI data. The model consists of an encoder and a decoder, and the input data is feature encoded and reconstructed through convolutional layers and upsampling layers. At the same time, we introduced a multi-sequence and multi-scale asynchronous correlation information extraction method to map the brain partition map under three-dimensional spatial coordinates to specific brain functional areas and extract the probability distribution of synchronous expression between different time series. The preprocessed numerical sequence data was state mapped by dynamic thresholding, and the dynamic correlation between time series was calculated. Finally, we used the extracted multi-scale asynchronous correlation information as the feature input model to train and construct an adolescent health risk prediction model.

### 3.1 2D convolutional autoencoder

An autoencoder (AE) is a neural network model primarily used for unsupervised learning. It achieves dimensionality reduction and feature extraction by learning to encode input data. The basic structure consists of two parts: the encoder and the decoder. The encoder maps the input data to a hidden representation, while the decoder attempts to reconstruct the original input from this hidden representation. By minimizing the error between the input data and the reconstructed data, the autoencoder learns useful features of the data.

We utilize a convolutional autoencoder model with the proposed number of layers to extract features from the Adolescent Health Risk Behaviors (AHRB) dataset (Demidenko M. I. et al., 2024). Initially, we preprocessed the raw data using the PyReliMRI toolkit (Demidenko M. et al., 2024), which includes standardized steps such as head motion correction, slice timing correction, and spatial normalization.

The goal of the autoencoder is to encode the input **x** into a hidden representation **h**, and then reconstruct the input **x** from **h**. The encoding and decoding processes are shown in Equations 1, 2, respectively:


(1) $$\begin{array}{l} \text{h}=f\left({\text{W}}_{1}\text{x}+{\text{b}}_{1}\right), \end{array}$$

(2) $$\begin{array}{l} \hat{\text{x}}=g\left({\text{W}}_{2}\text{h}+{\text{b}}_{2}\right), \end{array}$$

where **W**_1_ and **W**_2_ are the weight matrices of the encoder and decoder, respectively, **b**_1_ and **b**_2_ are the bias terms, and *f* and *g* are activation functions (typically nonlinear functions such as ReLU).

The loss function for the reconstruction error is typically the mean squared error (MSE), as shown in Equation 3:


(3) $$\begin{array}{l} L\left(\text{x},\hat{\text{x}}\right)=||\text{x}-\hat{\text{x}}|{|}^{2}, \end{array}$$

**Figure 2:**
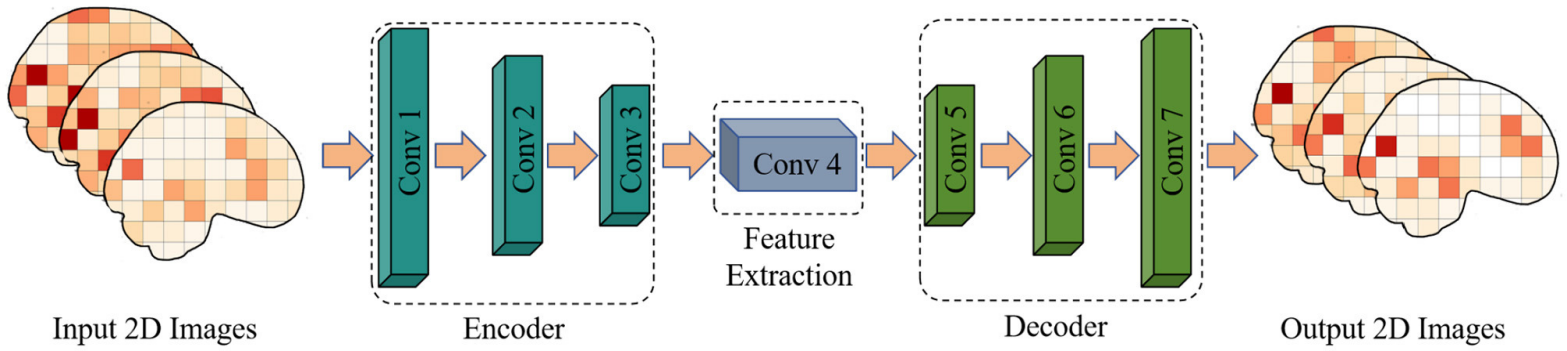
A 2DCNN-AE model for extracting features from fMRI images.

In the analysis of functional magnetic resonance imaging (fMRI) data, convolutional autoencoders can be used to extract spatial and temporal features for assessing adolescent health risks. The convolutional autoencoder comprises an encoder and a decoder. The encoder consists of three convolutional layers, each followed by a ReLU activation function and a max-pooling layer. The decoder consists of four convolutional layers, the first three layers are the upper sampling layer, and the last layer generates an image with the same shape as the input, as shown in Figure 2 and Table 1 for details.

**Table 1:** Depth information for our 2DCNN-AE model.

**Architecture**	**Layers**	**Output shape**		**Stride**	**Kernel size**	**Activation**	**Param**
		**Depth**	**Width**				
Encoder	Input layer	1	(96,96)	–	–	–	0
		Conv2D	24	(96,96)	1	24	ReLU	480
		Max pooling	24	(48,48)	1	–	–	0
		Conv2D	24	(48,48)	1	24	ReLU	8,046
		Max pooling	24	(24,24)	1	–	–	0
		Conv2D	1	(24,24)	1	24	ReLU	420
		Max pooling	1	(12,12)	1	–	–	0
Decoder	Conv2D	1	(12,12)	1	24	ReLU	10
		Upsampling	1	(24,24)	1	–	–	0
		Conv2D	24	(24,24)	1	24	ReLU	420
		Upsampling	24	(48,48)	1	–	–	0
		Conv2D	24	(48,48)	1	24	ReLU	8,046
		Upsampling	24	(96,96)	1	–	–	0
		Zero pad	1	(94,94)	–	–	–	0
		Conv2D	1	(94,94)	1	1	Tanh	480

For the input connectivity matrix data, features are first extracted through convolutional layers. The final layer of the encoder provides the hidden representation, as shown in Equation 4:


(4) $$\begin{array}{l} \text{h}=f\left({\text{W}}_{1}\text{x}+{\text{b}}_{1}\right), \end{array}$$

The hidden representation **h** is fed into the decoder, which reconstructs the input data through convolution and upsampling layers, as shown in Equation 5:


(5) $$\begin{array}{l} \hat{\text{x}}=g\left({\text{W}}_{2}\text{h}+{\text{b}}_{2}\right), \end{array}$$

Finally, the model optimizes its parameters by minimizing the reconstruction error, thereby learning useful features from the fMRI data for subsequent adolescent health risk assessment.

### 3.2 Multi-sequence multi-scale asynchronous correlation information extraction

**Figure 3:**
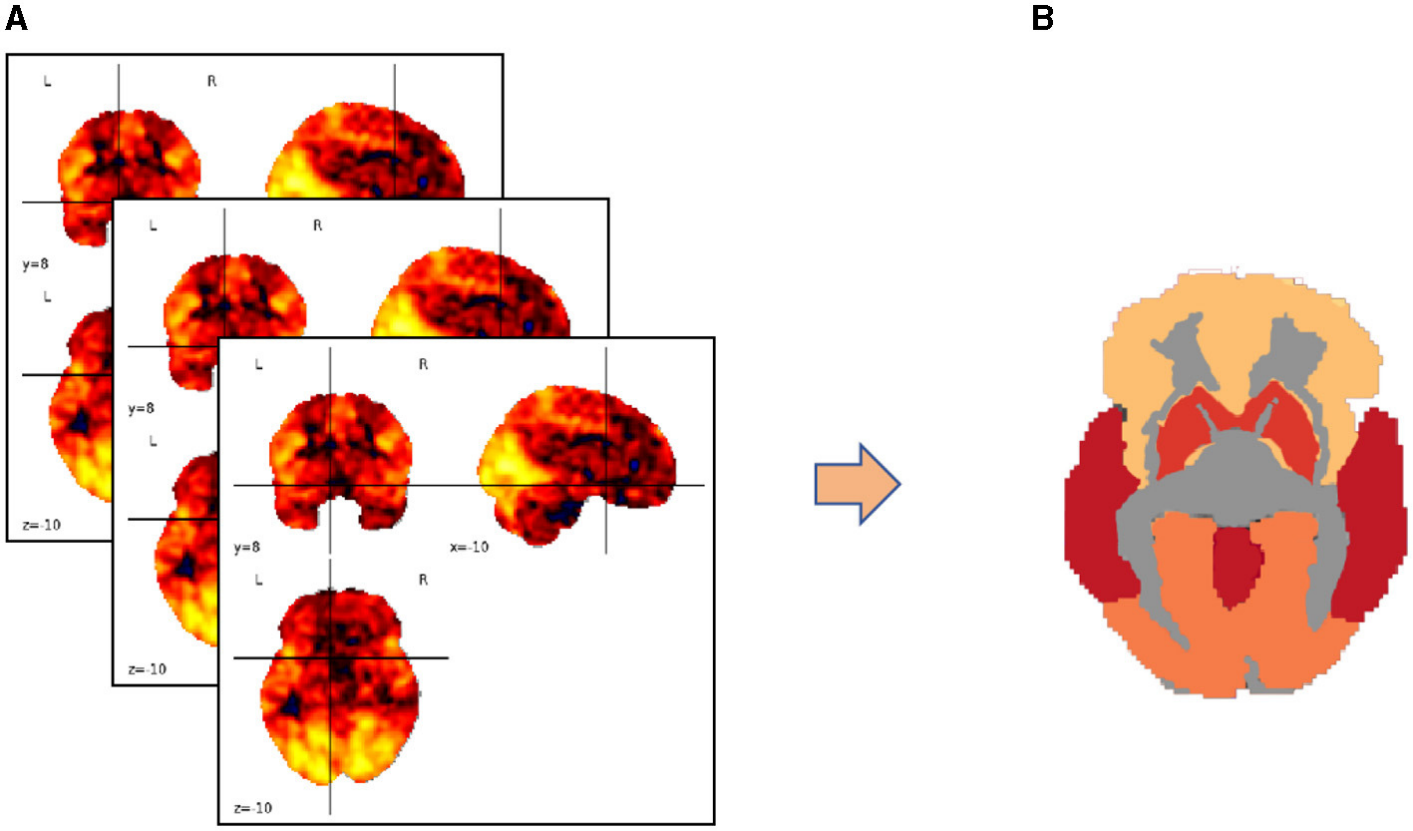
Transforming brain partition map targets into regions of interest (ROIs). **(A)** Three-dimensional coordinates of the brain partition map; **(B)** ROI distribution of fluctuation values of blood oxygen concentration levels in the brain area.

We map the voxels in three-dimensional spatial coordinates to specific brain functional regions using a brain partition map. The voxel values within each brain region are averaged to represent the overall fluctuation of blood oxygen concentration levels in that region, focusing our research on regions of interest (ROIs). This process is illustrated in Figure 3.

**Figure 4:**
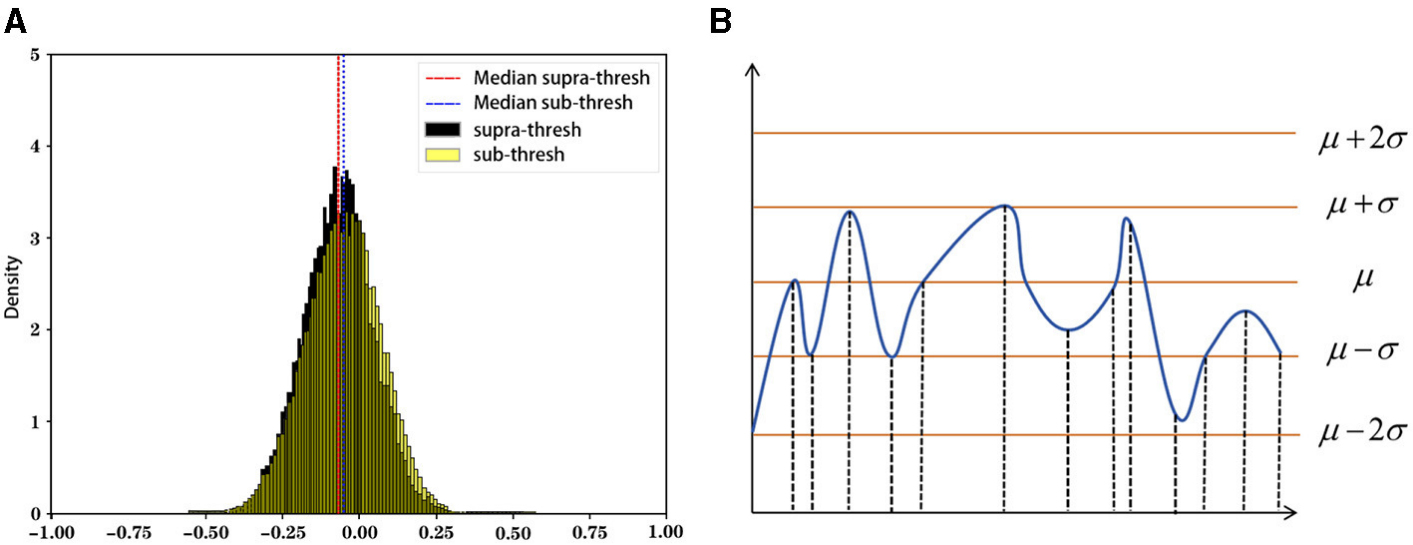
Rule of thumb and schematic diagram of state sequence mapping.

The state sequence mapping process converts the preprocessed numerical sequence data into state sequences. This study uses a dynamic threshold set by the rule of thumb (Figure 4), marking data above the threshold as active (1) and below as inactive (0). The threshold is defined as follows:


(6) $$\begin{array}{l} mT\left(H_{n}^{k},\eta \right)=\mu \left(H_{n}^{k}\right)+\eta \cdot \sigma \left(H_{n}^{k}\right), \end{array}$$

where $\mu \left(H_{n}^{k}\right)$ denotes the mean and $\sigma \left(H_{n}^{k}\right)$ denotes the standard deviation of the time series $H_{n}^{k}$, see Equations 7, 8 for details.


(7) $$\begin{array}{l} \mu \left(H_{n}^{k}\right)=\frac{\sum_{i=1}^{L}m_{n,l}^{k}}{\left|H_{n}^{k}\right|}, \end{array}$$

(8) $$\begin{array}{l} \sigma \left(H_{n}^{k}\right)=\frac{\sum_{i=1}^{L}{\left(m_{n,l}^{k}-\mu \left(H_{n}^{k}\right)\right)}^{2}}{|H_{n}^{k}|-1}, \end{array}$$

We define the mapping function $f\left(m_{n,l}^{k},\eta \right)$ to map each time slice η and $m_{n,m}^{k}$ to a state u:


(9) $$f(m_{n,l}^{k},\eta )=\{\begin{array}{l} \text{State}u_{1},\;m_{n,m}^{k}<mh(H_{n}^{k},\eta_{1})\\ \text{State}u_{2},\;mh(H_{n}^{k},\eta_{1})\leq m_{n,l}^{k}<th(H_{n}^{k},\eta_{2})\\ \cdots \\ \text{State}u_{s},\;mh(H_{n}^{k},\eta_{s})\leq m_{n,l}^{k}<mh(H_{n}^{k},\eta_{s+1})\\ \cdots \\ \text{State}u_{S},\;mh(H_{n}^{k},\eta_{S})\leq m_{n,l}^{k} \end{array},$$

### 3.3 Probability statistics of synchronous expression between brain regions

We extract discrete probability distributions of synchronous expression between brain regions. First, we define a function ϕ(·) to calculate the dynamic temporal relationship between two time series. The state sequence mapping step has successfully converted the fMRI data from a numerical sequence to a state sequence. Next, we use a measure function ψ(·) to evaluate the degree of synchronous expression between two time series, defined as in Equation 10:


(10) $$\begin{array}{l} \phi \left(m_{n_{1},l_{1}}^{k},m_{n_{2},l_{2}}^{k}\right)=\psi \left(f\left(m_{n_{1},l_{1}}^{k},\eta^{k}\right),f\left(t_{n_{2},l_{2}}^{k},\eta^{k}\right)\right), \end{array}$$

where *f*(·) represents the mapping function under certain prior conditions, converting data from a numerical sequence to a state sequence, and η^*k*^ denotes the mapping parameter. We then statistically analyze the frequency information of synchronous expression between brain regions. The function ψ(·) calculates the concurrent activation of two brain regions given a time slice parameter. This concurrent activation could be coincidental or indicative of potential interactions between these regions. With a sufficient number of time slices, we can obtain the probability distribution of whole-brain synchronous activity, distinguishing between coincidental and genuinely related phenomena, as shown in Equation 11:


(11) $$\begin{array}{l} \psi (f(m_{n_{1},l_{1}}^{k},\eta^{k}),f(m_{n_{2},l_{2}}^{k},\eta^{k}))\\ =\{\begin{array}{ll} 1, & m_{n_{1},l_{1}}^{k}>mh(H_{n}^{k},\eta^{k})\;and\;m_{n_{2},l_{2}}^{k}>mh(H_{n}^{k},\eta^{k})\\ 0, & else \end{array},\; \end{array}$$

For a multivariate time series data *H*_*k*_, the interaction between any two time series $H_{i}^{k}$ and $H_{j}^{k}$ is defined as follows in Equation 12:


(12) $$\begin{array}{l} X_{\phi \left(\cdot \right)}^{k}\left(i,j\right)=\overset{L}{\underset{i=1}{\sum }}\phi \left(m_{i,l}^{k},m_{j,l}^{k}\right), \end{array}$$

where *X*_ϕ(·)_(*i, j*) represents the cumulative sum of function values for each time slice pair. Based on this, the interaction between any two time series $H_{i}^{k}$ and $H_{j}^{k}$ is further defined with a time interval parameter *It* = [*rt, st*] as shown in Equations 13, 14:


(13) $$\begin{array}{l} X_{\phi \left(\cdot \right)}^{k}\left(i,j,I_{m}\right)=\overset{L}{\underset{i=1}{\sum }}\overset{s_{m}^{\prime }}{\underset{g=r_{t}}{\sum }}\phi \left(m_{i,l}^{k},t_{j,g+l}^{k}\right), \end{array}$$

(14) $$\begin{array}{l} s_{t}^{\prime }=\min\left(s_{t},M-m\right), \end{array}$$

This computes the interaction of custom asynchronous intervals with active-passive relationships. Note that $X_{\phi \left(\cdot \right)}^{k}\left(i,j,I_{m}\right)\neq X_{\phi \left(\cdot \right)}^{k}\left(j,i,I_{m}\right)$, indicating that the resulting interaction matrix *X*_ϕ(·)_ is asymmetric, as the interactions have active-passive relationships.

The comprehensive multivariate time series interactions, $X_{\phi \left(\cdot \right)}^{k}\in R^{N\times N\times T}$, represent multi-scale interval asynchronous synchronous expression values. Here, $X_{\phi \left(\cdot \right)}^{k}$ is a third-order tensor, where *N* denotes the number of time series, and *T* denotes the number of time slices of any time series. We convert the tensor $X_{\phi \left(\cdot \right)}^{k}$ into a discrete probability distribution form $Q_{\phi \left(\cdot \right)}^{k}$, defined as follows in Equation 15:


(15) $$\begin{array}{l} Q_{\phi \left(\cdot \right)}^{k}=\left\{q_{\phi \left(\cdot \right)}^{k}\left(i,j,I_{m}\right)|i,j\in \left[1,N\right],I_{m}\in I\right\}, \end{array}$$

where $q_{\phi \left(\cdot \right)}^{k}\left(i,j,I_{m}\right)$ denotes the discrete probability value between the *i*-th and *j*-th time series under interval parameter *I*_*m*_ and mapping function ϕ(·), defined as follows in Equation 16:


(16) $$\begin{array}{l} q_{\phi \left(\cdot \right)}^{k}\left(i,j,I_{m}\right)=\frac{X_{\phi \left(\cdot \right)}^{k}\left(i,j,I_{m}\right)}{\sum_{i=1}^{N}\sum_{j=1}^{N}\sum_{m=1}^{T}X_{\phi \left(\cdot \right)}^{k}\left(i,j,I_{m}\right)}, \end{array}$$

Finally, we use the extracted multi-scale asynchronous correlation information as features to train the model, resulting in an adolescent health risk prediction model.

## 4 Experiment

### 4.1 Dataset

The Adolescent Risk Behavior (AHRB) study dataset (Demidenko M. I. et al., 2024) recruited participants from diverse backgrounds to ensure a representative sample of the adolescent population. Each participant underwent a series of assessments, including neuroimaging, behavioral tests, and self-reported questionnaires. The primary focus of the study is to capture the dynamic changes in behavior and brain function as participants transition from late adolescence to early adulthood. The dataset includes two main cohorts from different years: Year 1 consists of approximately 108 participants aged 17–20, and Year 2 consists of approximately 66 participants aged 19–22. This study aims to track the developmental trajectory of risk behaviors and their underlying neural mechanisms.

The functional magnetic resonance imaging (fMRI) component of the AHRB study includes tasks designed to probe emotional and reward processing. Specifically, the study utilizes the Emotional Faces task and the Monetary Incentive Delay (MID) task. For our analysis, we use the raw Blood Oxygen Level-Dependent (BOLD) data from the MID task, which aligns with similar tasks used in the MLS and ABCD studies. The MID task requires participants to respond to cues indicating potential monetary rewards or losses. During the anticipatory phase, participants receive cues that indicate whether they can win or lose money based on their performance. The BOLD response during this phase is particularly interesting as it reflects the neural processes involved in anticipation, a critical component of reward-based decision-making. Understanding the neural basis of reward processing is crucial, as it is a key aspect of adolescent risk behavior.

### 4.2 Evaluation metrics

The evaluation metrics used in this study include accuracy (Acc), Precision (Prec), Recall (Rec), and F1−score. These metrics are defined as follows in Equations 17–20:


(17) $$\begin{array}{l} Acc=\frac{TP+TN}{TP+TN+FN+FP}, \end{array}$$

(18) $$\begin{array}{l} Prec=\frac{TP}{TP+FP}, \end{array}$$

(19) $$\begin{array}{l} Rec=\frac{TP}{TP+FN}, \end{array}$$

(20) $$\begin{array}{l} F1\text{-}score=\frac{2\times Prec\times Rec}{Prec+Rec}. \end{array}$$

Here, True Positive (TP) represents the correctly classified positive samples, True Negative (TN) represents the correctly classified negative samples, False Positive (FP) represents the incorrectly classified positive samples, and False Negative (FN) represents the incorrectly classified negative samples.

### 4.3 Model parameters

We first consider the threshold parameter η in Equation 6, which distinguishes the active or inhibitory states of the brain in fMRI imaging. A dynamic threshold converts a numerical sequence into a 0/1 sequence, where larger η values make the active state determination more stringent, resulting in fewer data points, while smaller values capture more data but may introduce noise. For all experiments, we set η = 1, as it balances avoiding overfitting while maintaining sufficient data points.

To improve model generalization given the limited data, we reduced the complexity of the 2DCNN-AE model by decreasing the number of layers and parameters in the convolutional layers. Additionally, we applied L2 regularization with a weight decay of 0.01 and used a Dropout rate of 0.5 in the fully connected layers to further prevent overfitting.

For model evaluation, we conducted a 10-fold cross-validation. The dataset was divided into 10 subsets, with the model trained on 9 subsets and validated on the remaining subset. This process was repeated 10 times, and the final performance metrics were averaged across all folds to ensure robust assessment of the model's stability and generalization. The experimental Settings are shown in Table 2.

**Table 2:** Experimental setup.

**Parameter**	**Value**
Threshold parameter (η)	1
L2 regularization	0.01
Dropout rate	0.5
Cross-validation	10-fold cross-validation
Dataset division	10 subsets (9 for training, 1 for validation), repeated 10 times
Time interval set (*I*)	[0, 0], [1, 1], [2, 2], [3, 6], [7, 12]

The primary goal of this study is to explore asynchronous functional connectivity between different regions of the adolescent brain. We extracted discrete probability distributions of synchronous expression at varying time intervals between brain regions. The interval set *I* was defined as [0, 0], [1, 1], [2, 2], [3, 6], [7, 12], with smaller intervals capturing short time delay interactions and larger intervals representing longer delays. This setup helps balance the sensitivity to synchronous information while minimizing the risk of overfitting due to excessively large delay parameters. The pseudocode for our algorithm is shown in Algorithm 1:

**Algorithm 1:**
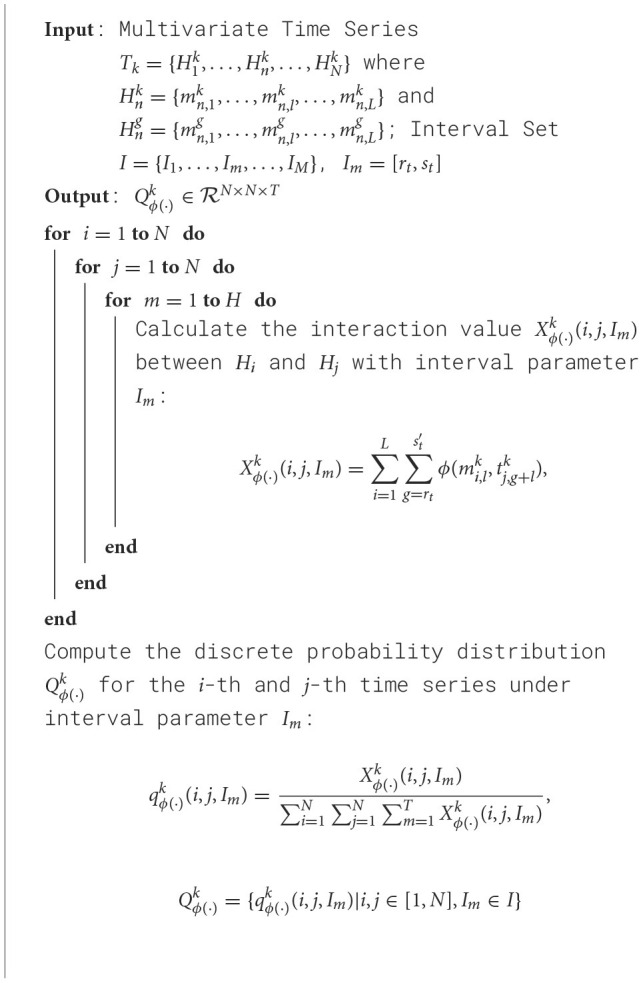
Training process of 2DCNN-AE net with multi-scale asynchronous correlation information extraction and synchronous expression probability statistics.

### 4.4 Experimental results

First, the experiment utilized a two-sample t-test as a feature selection method for dimensionality reduction. Next, the significance level parameter *p*-value was set to 0.05, 0.01, 0.005, and 0.001 respectively, with the results shown in Table 3.

**Table 3:** Feature reduction parameter validation results.

**Parameter(p)**	**Number**	**Acc(%)**	**Prec(%)**	**Rec(%)**	**F1-score(%)**
0.001	66	62.745	65.162	63.849	58.769
0.005	252	66.287	64.281	54.127	52.939
0.01	485	63.961	54.312	51.634	62.325
0.05	1102	65.254	61.361	64.395	62.841
0.1	1320	**70.142**	**66.276**	**71.946**	**68.995**
0.5	224	65.125	62.457	62.194	64.499

The best performance is shown in bold.

The results show that as the significance level parameter decreases, the number of extracted features also decreases. Initially, the experimental results improve with fewer features, but when the number of features is reduced too much (e.g., *p* = 0.001), the performance drops sharply. The best classification results were obtained with a significance level parameter of *p* = 0.005, achieving an accuracy, precision, recall, and F1-score of 70.142%, 66.276%, 71.946%, and 68.995%, respectively.

After selecting features with a significance level of *p* = 0.005, we further validated the parameter for the dynamic threshold μ in the state sequence transition. The experimental parameter μ was tuned within the range [0, 2] via grid search, with the results shown in Table 4.

**Table 4:** Dynamic threshold μ parameter validation results.

**μ**	**Acc(%)**	**Prec(%)**	**Rec(%)**	**F1-score(%)**
0.1	59.47	58.16	61.28	59.683
0.4	61.85	59.64	63.87	61.686
0.7	68.47	66.51	70.48	68.441
1.0	**70.81**	**68.64**	**73.41**	**70.950**
1.3	68.73	66.75	71.76	69.170
1.6	67.12	65.81	69.46	67.591
1.9	66.82	64.11	68.85	66.400
2.2	64.59	63.15	66.17	64.628
2.5	62.47	61.56	64.23	62.870
2.8	59.47	58.16	61.28	59.683
3.1	58.84	56.91	60.14	58.483
3.4	58.77	56.58	60.76	58.600
3.7	58.05	56.23	60.28	58.186
4.0	58.31	56.72	60.19	58.405

The best performance is shown in bold.

**Figure 5:**
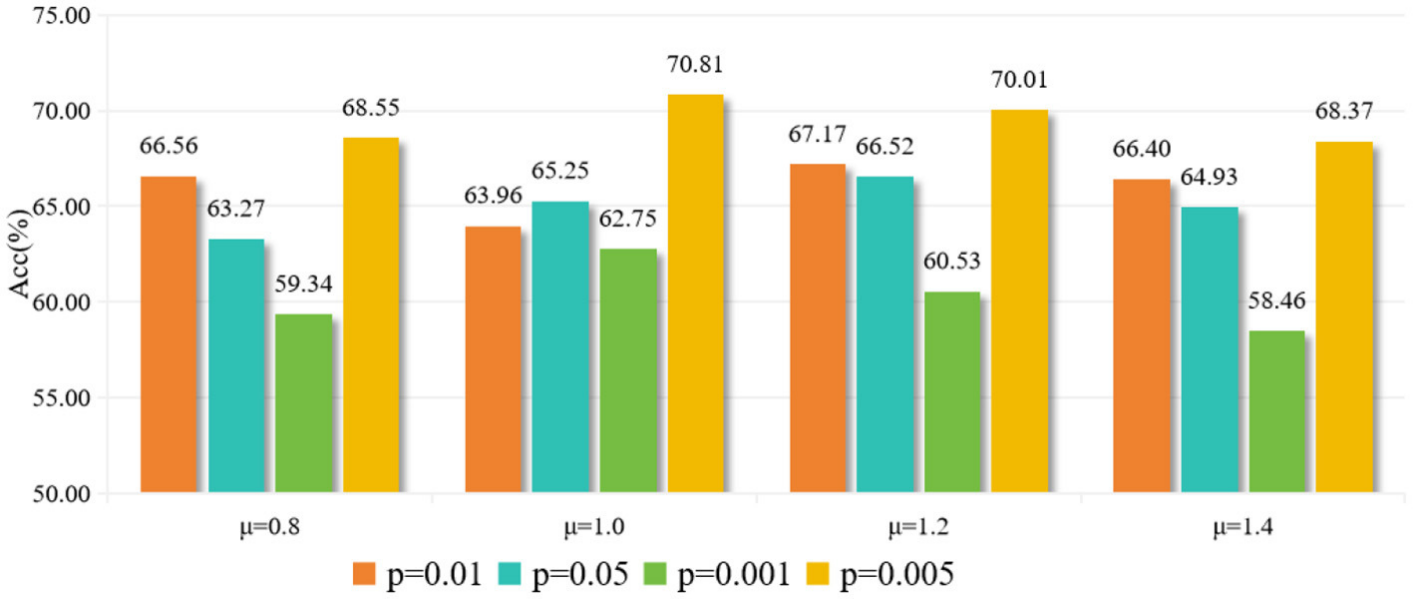
Experimental results with different parameter (*p*) values.

As shown in Table 4, using the full temporal mean value as the high activity threshold (μ = 0) yields poor results. This might be due to the low threshold being too broad, defining half of the time points as active, which introduces a lot of noise. As the threshold increases, the results improve. The best classification accuracy is achieved at μ = 1.0 and μ = 1.2, as a tighter definition of “active state” can effectively distinguish important activities. Beyond μ>1.2, the performance declines as the threshold becomes too high, leaving few points defined as active. The optimal default choice for the dynamic threshold parameter μ is 1.0. The experimental results for different parameters *p* are shown in Figure 5.

Next, we compare our model with methods using Pearson correlation coefficient, higher-order statistics (Wee et al., 2016), and dynamic functional connectivity models (Harlalka et al., 2019). Other popular methods include those by Zhang and Wang (2020), Brown et al. (2019), Abraham et al. (2017), and Yang et al. (2020). The results on the AHRB dataset are summarized in Table 5.

**Table 5:** Comparison with existing methods on the AHRB dataset.

**Method**	**Acc(%)**	**Prec(%)**	**Rec(%)**	**F1-score(%)**
Higher-order statistics	76.225	74.222	78.168	76.144
Zhang and Wang (2020)	80.051	82.450	80.577	81.503
Brown et al. (2019)	80.234	80.281	81.571	80.921
Yang et al. (2020)	78.061	82.052	81.222	81.635
Abraham et al. (2017)	**83.366**	82.775	83.665	83.218
Our	82.031	**83.116**	**84.784**	**83.942**

The best performance is shown in bold.

As shown in Table 5, our proposed method achieves precision, recall, and F1-score results of 83.116%, 84.784%, and 83.942%, respectively. Compared to the aforementioned methods, our method ranks highly in precision, recall, and F1-score. Although the accuracy is slightly lower than the method by Abraham et al. (2017) (83.366%), our method excels in the other three metrics. The experimental results demonstrate that our approach, incorporating 2D convolutional autoencoders and multi-sequence, multi-scale asynchronous information extraction, uncovers more asynchronous correlation information, yielding good classification accuracy in adolescent health risk assessment applications.

To validate our proposed improvements, we conducted three groups of ablation experiments. We added complex convolution and channel attention mechanisms to the 2DCNN network, along with phase smoothness and coil sensitivity smoothness as physical priors. The ablation experiments are summarized in Table 6, where coil sensitivity smoothness is denoted as S, phase smoothness as P, complex convolution as C, and channel attention mechanism as A. The baseline model is 2DCNN.

**Table 6:** Results of 2DCNN-AE network ablation experiments.

**S**	**P**	**C**	**A**	**Acc(%)**	**Prec(%)**	**Rec(%)**	**F1-score (%)**
				63.127	68.365	66.124	67.226
✓				69.125	63.452	68.045	65.668
	✓			70.058	68.716	70.098	69.400
✓	✓			75.858	72.383	72.881	72.631
		✓		72.015	71.228	70.365	70.794
			✓	75.365	75.824	75.339	75.582
		✓	✓	78.334	79.361	78.581	78.969
✓	✓	✓	✓	**81.031**	**83.116**	**84.784**	**83.942**

The best performance is shown in bold.

As shown in Table 6, each proposed improvement brought about performance enhancements. Individually adding coil sensitivity prior and phase prior led to considerable improvements, while the combination of channel attention mechanism and complex convolution resulted in significant gains. Combining all improvements achieved the best results.

These results indicate that the introduced 2D convolutional autoencoder and multi-sequence, multi-scale asynchronous information extraction methods effectively capture asynchronous correlation information, enhancing model performance in adolescent health risk assessment applications. The proposed modifications lead to significant improvements, as evidenced by the comprehensive ablation studies.

In summary, our method demonstrates superior performance in most metrics compared to existing methods, highlighting its potential in adolescent health risk assessment based on rs-fMRI data.

## 5 Discussion and conclusion

This study utilized fMRI and deep learning techniques to tackle challenges in adolescent health risk assessment, aiming to enhance the efficiency, accuracy, and interpretability of extracting features from fMRI data. We introduced a novel method integrating multi-sequence 2DCNN-AE with multi-scale asynchronous correlation information extraction, designed to capture spatial and temporal features and address the complex interactions between brain regions. Our experimental evaluation on the AHRB dataset demonstrated the method's superiority in accuracy, precision, recall, and F1-score, highlighting its capability to identify critical features and intricate temporal patterns often missed by traditional methods.

However, the study is not without limitations. First, the method's reliance on a relatively small dataset, due to the high cost and complexity of acquiring and processing fMRI data, may limit its generalizability to larger populations. This issue is particularly pronounced in deep learning models, which typically require large amounts of labeled data for effective training. Second, while the method improves interpretability compared to traditional deep learning approaches, the “black box” nature of certain deep learning components still poses challenges in clinical settings, where understanding the rationale behind predictions is crucial.

The superior performance of the proposed model can be attributed to its ability to capture both spatial and temporal dynamics from fMRI data. Specifically, the integration of multi-sequence 2DCNN-AE with multi-scale asynchronous correlation extraction allows for a more nuanced understanding of brain activity. This design choice helps to uncover latent interactions between brain regions that are otherwise overlooked in traditional models, leading to a more accurate assessment of health risks. Moreover, the asynchronous correlation extraction provides a mechanism to account for non-linear and time-shifted relationships between brain regions, which may be critical in identifying early indicators of health risks. These insights not only demonstrate the efficacy of the proposed approach but also open new avenues for exploring brain region connectivity in health-related research.

Looking forward, future research should focus on addressing these limitations. Expanding the dataset size through collaborative efforts and leveraging transfer learning techniques could help improve the model's generalizability. Additionally, integrating more interpretable machine learning methods or developing hybrid models that combine deep learning with rule-based systems could further enhance the clinical applicability of the proposed method. These improvements would not only increase the accuracy and robustness of the predictions but also make them more actionable for healthcare providers.

In conclusion, the superior performance of the proposed model can be attributed to its ability to capture both spatial and temporal dynamics from fMRI data. Specifically, the integration of multi-sequence 2DCNN-AE with multi-scale asynchronous correlation extraction allows for a more nuanced understanding of brain activity. This design choice helps to uncover latent interactions between brain regions that are otherwise overlooked in traditional models, leading to a more accurate assessment of health risks. Moreover, the asynchronous correlation extraction provides a mechanism to account for non-linear and time-shifted relationships between brain regions, which may be critical in identifying early indicators of health risks. These insights not only demonstrate the efficacy of the proposed approach but also open new avenues for exploring brain region connectivity in health-related research.

## Data Availability

The datasets presented in this study can be found in online repositories. The names of the repository/repositories and accession number(s) can be found in the article/supplementary material.
